# Construction Notes

**Published:** 1896-04-11

**Authors:** 


### CONSTRUCTION NOTES.

### New Cottage Hospital at Ormskirk.

This building just erected at Ormskirk will be a great benefit to the district. It consists of three blocks on the pavilion system, connected by a corridor 55 ft. long. The central block consists of entrance lobby, operating-room, and bath-room, and a ward for two beds on the south side. This ward is dedicated to the late Dr. Ryder. At either end of the corridor is a ward, one for males and the other for females, each with space for three beds. The sanitary blocks are disconnected from the wards by ventilated lobbies. The three wards face south, and are so arranged that the accommodation can be increased at a minimum of cost. The floors are of red pine, oil-stained and varnished. The warming and ventilation of the wards are by Manchester and ordinary open grates and iron fresh-air pipes, which can be removed for cleaning. In the corridor are the coils of hot-water pipes, which are heated by means of a Halifax independent boiler. This also provides the hot-water supply throughout the building. Externally the building is built of grey St. Helens brick, with red brick dressings. The whole of the drainage has been planned and executed in the most modern manner, and easy access and ample ventilation to all parts has been provided. The contractor was Mr. James Pilkington, of Rainford; the architect, Mr. C. S. Beeston, of Ormskirk, M.S.I., of London.

